# Integration of structural brain networks is related to openness to experience: A diffusion MRI study with CSD-based tractography

**DOI:** 10.3389/fnins.2022.1040799

**Published:** 2022-12-08

**Authors:** Nima Talaei, Amirhossein Ghaderi

**Affiliations:** ^1^Department of Psychology, Faculty of Literature and Human Sciences, Shahid Bahonar University, Kerman, Iran; ^2^Hotchkiss Brain Institute, University of Calgary, Calgary, AB, Canada; ^3^Department of Psychology, University of Calgary, Calgary, AB, Canada

**Keywords:** openness to experience, personality, structural connectivity, diffusion MRI, CSD-based tractography, graph theory

## Abstract

Openness to experience is one of the big five traits of personality which recently has been the subject of several studies in neuroscience due to its importance in understanding various cognitive functions. However, the neural basis of openness to experience is still unclear. Previous studies have found largely heterogeneous results, suggesting that various brain regions may be involved in openness to experience. Here we suggested that performing structural connectome analysis may shed light on the neural underpinnings of openness to experience as it provides a more comprehensive look at the brain regions that are involved in this trait. Hence, we investigated the involvement of brain network structural features in openness to experience which has not yet been explored to date. The magnetic resonance imaging (MRI) data along with the openness to experience trait score from the self-reported NEO Five-Factor Inventory of 100 healthy subjects were evaluated from Human Connectome Project (HCP). CSD-based whole-brain probabilistic tractography was performed using diffusion-weighted images as well as segmented T1-weighted images to create an adjacency matrix for each subject. Using graph theoretical analysis, we computed global efficiency (GE) and clustering coefficient (CC) which are measures of two important aspects of network organization in the brain: functional integration and functional segregation respectively. Results revealed a significant negative correlation between GE and openness to experience which means that the higher capacity of the brain in combining information from different regions may be related to lower openness to experience.

## Introduction

One of the main goals of personality psychology is to provide valid taxonomies of human personality that can describe how individuals differ from one another in behavior, motivation, emotion, and cognition. The Five-Factor Model (FFM) is the most widely used of these taxonomies which describes individual differences as a consequence of variation in five big traits: openness to experience, conscientiousness, extraversion, agreeableness, and neuroticism (Costa and McCrae, 1992a,b). Openness to experience can be defined as one’s tendency to seek, appreciate, comprehend, and utilize information; It represents the psychological function of cognitive exploration and usually shows a positive correlation with IQ test performance (DeYoung et al., 2014; DeYoung, 2015). In addition, it is the best predictor of mind wandering and creativity, among the five big traits (Karwowski and Lebuda, 2016; Ibaceta and Madrid, 2021). Heightened openness to experience has also been linked to psychosis proneness and some have speculated that there might be common biological underpinnings between openness and psychosis (DeYoung and Krueger, 2018; Blain et al., 2020). Hence understanding the neurocognitive mechanisms of openness to experience may also help in understanding its relationship with psychosis. But debates remained unresolved about the neural origins that cause individual differences in openness to experience. Identifying neural correlates of the five-factor model (including openness to experience) is a major subfield in personality neuroscience and can have significant implications for understanding the biological factors involved in different cognitive and behavioral tendencies (DeYoung, 2015, p. 4). Personality neuroscience is a relatively new field of study that seeks to find the neural underpinnings of personality and therefore is an attempt to provide insight into the causal mechanisms of individual differences (DeYoung, 2010). It allows researchers to investigate the brain mechanisms underlying each trait using a wide variety of techniques. Among different techniques that are used in personality neuroscience, magnetic resonance imaging (MRI) is the most frequently used method due to its versatile nature (Allen and DeYoung, 2016). MRI provides great opportunities and also interesting challenges in understanding the neural correlates of openness to experience.

Various MRI studies have been done on structural brain regions that are associated with the trait of openness to experience. Some studies investigated the relationship between Fractional Anisotropy (FA) and Mean Diffusivity (MD) of various white matter tracts with openness to experience. Both increased FA and decreased MD may reflect greater white matter microstructural organization. Openness has been reported to have a positive correlation with FA values in the white matter adjacent to the dorsolateral prefrontal cortex (DLPFC) in both hemispheres while having a negative correlation with the mean diffusivity (MD) of the same regions which show a relationship between higher openness to experience and greater white matter microstructural organization in the aforementioned regions (Xu and Potenza, 2012). Later research partly replicated these results and found a positive correlation between openness to experience and FA values in inferior frontal-occipital fasciculus (IFO) and inferior longitudinal fasciculus (ILF) (Privado et al., 2017). Both of these studies used diffusion tensor imaging (DTI) and had similar sample sizes (∼50 Participants), but there was only partial overlap between the white matter tracts that were analyzed which can explain the failure in replication of all the results of the former study by the latter (Xu and Potenza, 2012; Privado et al., 2017). However, three other studies found no correlation between openness to experience and any of the DTI measures in healthy individuals (Bjørnebekk et al., 2013; Rodriguez et al., 2019; Avinun et al., 2020). The inconsistency between the findings of these five studies might be due to factors like effect size, multiple comparisons, or the use of different methods in analyzing diffusion data. This inconsistency can be observed in morphometric studies as well. Owens et al. (2019) conducted a study based on the Human Connectome Project (HCP) data (*n* = 597) and using the FreeSurfer software package to investigate the neuroanatomical correlates of the big five traits and reported that both cortical thickness and volume of several regions are associated with openness to experience. However, a recent study with the largest sample of any personality neuroscience study to date (*n* = 1,107), failed to replicate these FreeSurfer results (Avinun et al., 2020).

Since these results cannot be merged into a global framework, we may need a more comprehensive view to consider all these involved areas as structural/functional brain networks. Accordingly, the study of the neural underpinnings of personality can be advanced by moving beyond accounts that are solely based on the structure and function of discrete brain regions and subsequently adding a network perspective on brain structure and function (Markett et al., 2018). Some functional studies have already tried to investigate the relationship between openness to experience and functional connectivity in resting-state networks (Beaty et al., 2018; Wang et al., 2018, 2022; Marstrand-Joergensen et al., 2021) and some of these studies support the existence of a positive association between openness to experience and functional connectivity within and between resting state networks. However, these studies did not evaluate the architecture and topology of brain networks that are constructed by a huge number of connections between all pairs of brain regions. To investigate these complex network architectures, we need more advanced approaches in network science like graph-theoretical analysis (Bullmore and Sporns, 2009).

During the last 15 years, significant progress has been made in the application of graph theory in analyzing the connectome. Connectome is a network map of brain connectivity that can be constructed from structural (e.g., diffusion MRI data) or functional data (e.g., functional MRI data). This network map consists of nodes (brain regions) and edges (connectivity). It can be summarized in the form of a connectivity (adjacency) matrix (Sporns et al., 2005; Bullmore and Sporns, 2009), and to analyze these connectivity matrices and extract topological features of associated brain networks, graph theoretical analysis is used. In this framework, two important topological network measures which illustrate the architecture of connection in the local and global circuits are the clustering coefficient and global efficiency, respectively (Ghaderi et al., 2018, 2020). The clustering coefficient, which is sensitive to the topology of local and modular circuits, reveals brain network *segregation* and global efficiency which is associated with the capacity of the network in combining the neural signals from different regions, disclosing brain network *integration* (Rubinov and Sporns, 2010; Ghaderi et al., 2018, 2020).

Graph theoretical analysis has been used in some studies investigating the relationship between personality and functional connectome (Beaty et al., 2016; Toschi et al., 2018; Cai et al., 2020), notably Toschi et al. (2018) assessed the relationship between the big five traits and the different graph measures gained from functional connectome in a functional MRI data sample from Human Connectome Project (HCP). They reported a positive association between conscientiousness and graph measures of local connectivity, namely the local clustering coefficient in the frontoparietal network (FPN) and default mode network (DMN) but found no association between openness to experience and any graph measure (Toschi et al., 2018). Much fewer studies so far incorporated the graph theoretical analysis into the investigation of the structural connectome of the big five traits. Ueda et al.’s (2018) study is the only study that we know of that applied graph theoretical analysis to investigate the association between one of the big five traits (neuroticism) and graph measures gained from structural connectome (Ueda et al., 2018). They found a significant relationship between neuroticism and graph measure of betweenness centrality in several regions but did not find any relationship between other graph measures and neuroticism. Hence it remains to be determined if there is a relationship between various graph measures gained from structural connectome and openness to experience. Understanding the relationship between openness to experience and structural connectome is not only important in the face of heterogeneous findings of previous studies, but also can provide a more comprehensive view of the neural underpinnings of openness to experience and its possible link with psychosis.

The current study aims to examine the relationship between openness to experience and structural brain network *integration* and *segregation*. To our knowledge, no study has investigated the relationship between openness to experience and graph measures gained from structural connectome to date. Since previous studies found inconsistent results and reported associations between various structural brain regions and openness to experience, we decided to use structural connectome analysis to investigate if there is a relationship between structural brain network features and openness to experience. Our study benefits from state-of-the-art High Angular Resolution Diffusion Imaging data (HARDI) from HCP (Van Essen et al., 2012b; Sotiropoulos et al., 2013) which is ideal for the multi-tissue Constrained Spherical Deconvolution (CSD) modeling (Jeurissen et al., 2014). CSD-based probabilistic tractography has been indicated to be a superior method in white matter fiber tractography compared to DTI-based tractography (Farquharson et al., 2013; Auriat et al., 2015).

## Materials and methods

### Subject’s data

The minimally pre-processed structural and diffusion MRI data along with the openness to experience trait score from self-reported NEO-FFI of 100 subjects (50 men and 50 women; aged from 22 to 35) were randomly selected and downloaded from the HCP repository. Currently, the HCP repository contains MRI, and personality data of 973 subjects, and since all HCP subjects are healthy young adults (aged from 22 to 37), our only inclusion criterion in the selection of subjects was the equal number of men and women (Jenkinson et al., 2002, 2012; Glasser and Van Essen, 2011; Marcus et al., 2011; Fischl, 2012; Van Essen et al., 2012b; Glasser et al., 2013; Elam et al., 2021). Due to the limited computational power that we had access to as well as the time restrictions of the current study, we were not able to analyze the whole data set, but we aimed for a sample size that is larger than many similar studies (Xu and Potenza, 2012; Privado et al., 2017; Ueda et al., 2018). The data is publicly available at the HCP repository and the list of subjects’ IDs whose data were analyzed in the current study is reported in the Supplementary material.

### Personality assessment

The sum of scores of openness to experience factor from the NEO five-factor inventory (NEO-FFI) was used as a measure of openness trait for each subject (Costa and McCrae, 1992c). It is the shortened version of NEO-PI-R which has 240 Items (Costa and McCrae, 1992c). NEO-FFI consists of 60 Items: 12 Items for each of the five dimensions of personality. It assesses each factor on a five-point Likert scale that is ranged from strongly agree to strongly disagree. For each personality trait, scores range from 0 to 48, and in the case of openness to experience, a higher score represents a higher tendency to seek, appreciate, comprehend, and utilize information (DeYoung, 2015). NEO-FFI has shown excellent reliability and validity; Cronbach’s alpha ranges from 0.75 to 0.82 for the five scales and 0.76 for openness to experience (McCrae and Costa, 2004; McCrae et al., 2005).

### Magnetic resonance imaging scanning protocol

Diffusion-weighted MRI is an imaging modality that can be used to examine the anatomical connectivity and tissue microstructure, by measuring the local diffusion of water molecules (O’Donnell and Westin, 2011). Even though MRI is an indirect method of measuring the structure and function of the brain and MRI images are subject to noise, during the last few years significant progress has been made in multiband imaging techniques and image processing methods especially in the context of human connectome project (HCP) (Feinberg et al., 2010; Moeller et al., 2010; Setsompop et al., 2012; Sotiropoulos et al., 2013; Uǧurbil et al., 2013), resulting in substantial improvement of spatial and temporal resolution of images (Shi and Toga, 2017; Schilling et al., 2018).

The MRI data were acquired on a customized Siemens Magnetom Skyra 3T MRI system using a multi-band pulse sequence. Compared to a standard Skyra MRI, the customized hardware has a higher gradient strength which benefits diffusion imaging (Feinberg et al., 2010; Uǧurbil et al., 2013). The parameters selected for the diffusion-weighted imaging include 270 diffusion-weighted volumes equally distributed over three shells of *b* = 1,000, 2,000, and 3,000 s/mm^2^ and a total of 18 reference volumes (*b* = 0 s/mm^2)^, voxel size of 1.25 mm isotropic, 174 slices, 145 × 145 matrix, and TR/TE = 5,520/89.5 ms. HCP High-resolution T1-weighted anatomical images (T1w) were acquired using the 3D magnetization prepared rapid gradient echo sequence (MPRAGE) (Mugler and Brookeman, 1990) with a voxel size of 0.7 mm^3^, TR/TE = 2,400/2.14 ms, and flip angle of eight degrees. The T1w images from all participants were subject to a standard quality control process, including manual viewing and rating of quality by an experienced rater. However, performing comprehensive quality control for all diffusion MRI (dMRI) and functional MRI (fMRI) scans was not feasible, and in the interest of providing data, dMRI and fMRI scans were rarely excluded for motion and hence quality issues may be present with some scans. For more details about the data acquisition, preprocessing pipeline, and image quality control, refer to the following papers (Van Essen et al., 2012a,b; Marcus et al., 2013; Sotiropoulos et al., 2013; Elam et al., 2021).

### Estimation of structural connectivity

We followed the framework proposed by Civier et al. (2019) in the estimation of structural connectivity using HCP Data (Civier et al., 2019). The first step in processing the already minimally preprocessed HCP diffusion data (Andersson et al., 2003; Glasser et al., 2013; Andersson and Sotiropoulos, 2015, 2016) was biased field correction using Advanced Normalization Tools (ANTs) (Tustison et al., 2010). To process the diffusion data and model the white matter, gray matter, and cerebrospinal fluid (CSF), we used the MRtrix3 software package^1^ (Tournier et al., 2019). Since the HCP data is collected with multiple shells, we used the multi-shell multi-tissue constrained spherical deconvolution (CSD) method (Jeurissen et al., 2014) to generate Fiber Orientation Densities (FODs) (Tournier et al., 2004, 2007). We then performed Multi-tissue informed log-domain intensity normalization (Dhollander et al., 2021). Next, the T1w anatomical images got segmented using FMRIB Software Library^2^ (version 6.0.1) (Jenkinson et al., 2012) and co-registered with diffusion-weighted images to create tissue boundaries needed to perform the subsequent probabilistic tractography using MRtrix3 (Tournier et al., 2004). A total of 10 million probabilistic streamlines were generated using Second-order Integration over Fiber Orientation Distributions (iFOD2) (Tournier et al., 2010) and Anatomically-Constrained Tractography framework (ACT) (Smith et al., 2012). ACT ensures that streamlines that terminate in CSF will be dismissed. In other words, ACT constrains streamlines to the white matter and therefore makes them biologically more plausible (Smith et al., 2012). Other parameters that were used in tractography are dynamic seeding [which determines seed points dynamically using the SIFT model (Smith et al., 2015a)], backtracking (an option that allows tracks to be truncated and re-tracked if a poor structural termination is encountered), FOD amplitude threshold of 0.06, step size of 0.625 mm, and length of 5–300 mm (Civier et al., 2019).

It is a known issue in neuroimaging that the number of streamlines connecting different gray matter regions as a measure of connection density is not a valid representation of axonal count (Jones et al., 2013). Tractogram reconstruction can introduce several biases that make the streamline count not completely reflective of the underlying white matter structure (Jones et al., 2013). Therefore, the second version of Spherical-deconvolution Informed Filtering of Tractograms (SIFT2) was used as a method to reduce these biases by selectively removing some of the streamlines and as a result, creating a more biologically meaningful measure of structural connectivity (Smith et al., 2015b).

For each subject, regions of interest (ROIs) were selected based on an already segmented T1w anatomical image using FreeSurfer and the Desikan-Killiany atlas (Desikan et al., 2006; Fischl, 2012). This particular atlas is used in several personality studies (Ueda et al., 2018; Vartanian et al., 2018; Delaparte et al., 2019; Avinun et al., 2020). All 84 ROIs of the segmented images were chosen in order to create whole-brain structural connectomes for the subsequent graph analysis. For every subject, a whole-brain zero diagonal adjacency matrix (84 × 84) was constructed where each array presented structural connectivity between the corresponding row and column. The value of each array was computed by summing the weights of the relevant streamlines. Weighted matrices were used for subsequent graph analysis.

### Graph theoretical analysis

We computed two measures of complex networks: *global efficiency* (GE) and *clustering coefficient* (CC). These two measures characterize topological aspects of whole brain networks by presenting two important features, i.e., the integration of all neural pathways and the segregation of local modules, respectively. Many studies have suggested that these two features are correlated with different aspects of brain functions, cognition, and perception (Rubinov and Sporns, 2010; Fischer et al., 2014; Masuda et al., 2018; Ghaderi et al., 2018, 2020). Although other topological measures of the whole brain network, like characteristic shortest path and transitivity, can also be used to assess these brain features, since these measures are mathematically related to *GE*, and *CC* (shortest path is the inverse of global efficiency and transitivity has only coefficient difference with *CC*), we avoid calculating them. Functional integration in the brain is the capacity of the brain in combining information from different regions. It can also be viewed as the ability of the brain network for efficient global communication (Rubinov and Sporns, 2010). Several studies show that structural brain networks can be considered highly integrated (Bullmore and Sporns, 2009; Park and Friston, 2013). Mathematically, GE is the average over the inverse shortest path length between all pairs of nodes in a network (Latora and Marchiori, 2001):


$$G\,E=\frac{1}{n}\,\sum_{i\in N}E_{i}=\frac{1}{n}\,\sum_{i\in N}\frac{\sum_{j\in N,j\neq i}d_{i\,j}^{-1}}{n\;-\;1}.$$

Where *N* is the set of all nodes in the network, *n* is the number of nodes, and *i, j* are indices of nodes. *d*_*ij*_ is the shortest path length (distance) between nodes *i* and *j* while *E*_*i*_ is the efficiency of node *i*. In the structural brain networks, these paths represent white matter pathways and the existence of shorter pathways can suggest higher GE and therefore stronger functional integration in the brain (Rubinov and Sporns, 2010).

Functional segregation is the capacity of the brain to process information in local circuits (modules). Consequently, in the human brain and the nervous system of many other species, high functional segregation is the result of a large number of modules’ existence (Rubinov and Sporns, 2010). Structural modules provide two main functionalities: first, they provide an efficient sharing of information among sets of brain regions. second, they promote functional specialization by limiting the boundaries of each module and preventing the spread of information across the whole network (Rubinov and Sporns, 2010; Sporns, 2016). CC is defined based on the number of triangles (the node’s neighbors that are also neighbors of each other) that a node creates with its neighbor nodes. Mathematically, CC is calculated by Watts and Strogatz (1998):


$$C\,C=\frac{1}{n}\,\sum_{i\in N}C_{i}=\frac{1}{n}\,\sum_{i\in N}\frac{2\,t_{i}}{k_{i}\,\left(k_{i}-1\right)}.$$

Where *k*_*i*_ is the degree of node *i*, *t*_*i*_ is the number of triangles around the node *i* and *C*_*i*_ is the CC of node *i* (*C*_*i*_ = 0 for *k*_*i*_ < 2). The CC of all nodes can be used as a measure of segregation of the whole network (Rubinov and Sporns, 2010).

The network measures of all 100 brain networks were computed using Brain Connectivity Toolbox (BCT)^3^ (Rubinov and Sporns, 2010) in MATLAB. The CC was computed for each node, then the average CC of all nodes was calculated for each subject. The GE of the whole brain network was computed for each subject as well.

### Statistical analysis

We performed linear and non-linear analysis using MATLAB 2021b to evaluate the association of each network measure (GE and CC) with the openness score. The linear regression model was performed using the *fitlm* function twice: first to evaluate the relationship between GE and the openness trait score. Second, to investigate the association between CC and the openness trait score. But before performing each regression analysis, we calculated the Cook’s distance to detect outliers that might have been affected by noise during image acquisition and/or had an unusual openness score. Cook’s distance is a method that is useful for identifying outliers usually in the context of regression analysis. It takes into account both the leverage and residual of each observation. We used the *plotDiagnostics* function to detect observations with Cook’s distance larger than three times the mean Cook’s distance. A total of ten subjects got removed from the first analysis and nine subjects got removed from the second analysis due to having cook’s distances of more than three times the mean (Cook, 1977; Ueda et al., 2018). Next, we corrected for multiple comparisons (type one error) to correct for the number of network measures using Bonferroni-Holm correction, and corrected *p*-values less than 0.05 were considered significant (Holm, 1979; Cho and Martinez, 2014).

**FIGURE 1:**
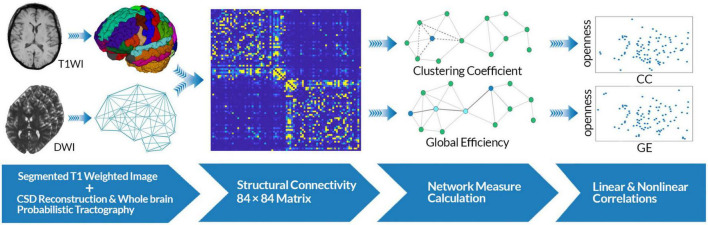
Data analysis workflow. Constrained spherical deconvolution (CSD)-based whole-brain probabilistic tractography was performed using diffusion-weighted images as well as segmented T1w images. T1w images were segmented based on the Desikan-Killiany atlas for each subject. A whole-brain adjacency matrix (84 × 84) was constructed for each subject. Graph theoretical analysis was performed on each matrix to compute global efficiency (GE) and clustering coefficient (CC) as measures of integration and segregation in the brain structural networks. Linear and non-linear analyses were performed to evaluate the association between each network measure and openness score.

We then conducted two linear and non-linear analyses, namely Linear Regression and Gaussian Process Regression (GPR) using the *Regression learner Tool* in MATLAB to see how accurately graph measures can predict openness scores using each of these two models. The regression learner tool allows the user to train and validate regression models using different algorithms and compare results to find the best model. In general, different supervised learning algorithms (linear regression and GPR among them) try to solve the problem of input-output mapping from the training dataset using various approaches. In other words, by estimating a function based on the training data, they make predictions for new inputs that were not part of the training data. The problem is that for any given data set, there are potentially an infinite number of functions that can fit it (Rasmussen and Williams, 2016). One approach to solve this problem is to only consider a strict class of functions, for example by only considering linear functions of the input. GPR, as a non-parametric Bayesian approach to regression, provides a different solution for the aforementioned problem, which is allocating a probability to each of the estimated functions based on prior knowledge (kernels). The mean of the resulting probabilistic distribution represents the most probable description of the data (Rasmussen and Williams, 2016). Consequently, GPR is one of the most versatile non-linear methods in the field of machine learning and recently has been widely used in neuroscience and psychology (Caywood et al., 2017; Bahg et al., 2020). We compared results from GPR with an exponential kernel, with the linear regression model results to see if a non-linear approach can better describe our data compared with a linear model. We used cross-validation rather than hold-out validation, as the latter requires a very large dataset to correctly function and the former is a powerful preventative measure against overfitting, especially in relatively small datasets. In cross-validation, the dataset gets partitioned into k groups (i.e., k-fold). One of the groups is then used as a test set and k-1 groups are used as the training set. The process of training and testing is then repeated until each group is used as the test set, which substantially reduces the over-fitting problem (Browne, 2000; Yadav and Shukla, 2016). We did a five-fold cross-validation which means that in each test, 80% of the data is used for the training while 20% is used for testing. The complete data analysis workflow is summarized in Figure 1. To evaluate the accuracy of prediction for each model, we reported root mean square error (RMSE) and accuracy:


$$\mathrm{Accuracy}=\left(1-\frac{\left(P_{\mathrm{Real}}\;-\;P_{\mathrm{predicted}}\right)}{P_{\mathrm{Real}}}\right)\times 100$$

Where *P*_*Real*_ is the real parameter and *P*_*Predicted*_ is the predicted parameter.

## Results

**FIGURE 2:**
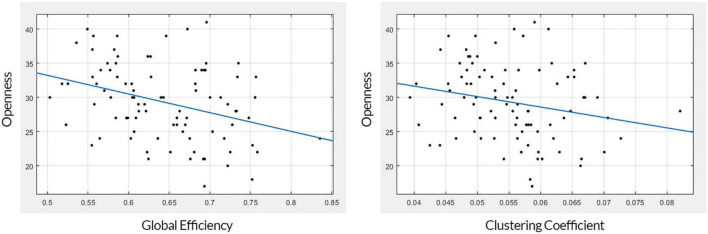
Linear correlation plots. The plot on the left shows the association between global efficiency (x-axis) and openness to experience (y-axis). The plot on the right shows the association between the clustering coefficient (x-axis) and openness to experience (y-axis).

The mean and standard deviation of openness to experience scores before and after outlier removal is presented in Table 1. The standard deviation of openness to experience scores decreased by approximately 0.6 after outlier removal for each regression analysis. The GE and CC measures gained from the structural brain networks were initially both significantly associated with openness to experience scores (GE: *r* = −0.344, *F* = 11.8, *p* = 0.0008; CC: *r* = −0.222, *F* = 4.65, *p* = 0.0337). After correcting for multiple comparisons using Bonferroni correction, GE still had a moderate negative association with the openness scores (*p* = 0.001), but the CC scores were not (*p* = 0.067) (see Table 2). Figure 2 presents linear correlation plots between openness to experience scores and CC and GE. Results from the statistical analysis without outlier removal are presented in the Supplementary material.

**TABLE 1:** The mean and standard deviation of openness to experience, GE and CC scores before and after outlier removal using the cook’s distance method.

State of outliers	Number of subjects	Openness scores		GE scores		CC scores	
		Mean score	Standard deviation	Mean score	Standard deviation	Mean score	Standard deviation
Without outlier removal	100	29.20	6.06	0.6414	0.0765	0.0551	0.00800
GE and openness regression outlier removal	90	29.30	5.49	0.6441	0.0693	–	–
CC and openness regression outlier removal	91	29.29	5.45	–	–	0.0554	0.00795

**TABLE 2:** The association between openness to experience scores and two graph measures of global efficiency and clustering coefficient using linear regression.

Graph measure	Pearson’s *r*	*R* ^2^	Adjusted *R*^2^	RMSE	Standard error	*F*-statistic	*P*-value	Corrected *P*-value	Number of subjects
Global efficiency	−0.3443	0.119	0.109	5.19	7.939	11.8	0.0008*	0.0016*	90
Clustering coefficient	−0.2229	0.049	0.039	5.34	70.797	4.65	0.0337*	0.0674	91

*Indicates the *p*-values that were statistically significant (*p* < 0.05). RMSE, root mean square error; Corrected *p*-value = The *p*-value after multiple comparisons using Bonferroni correction.

**FIGURE 3:**
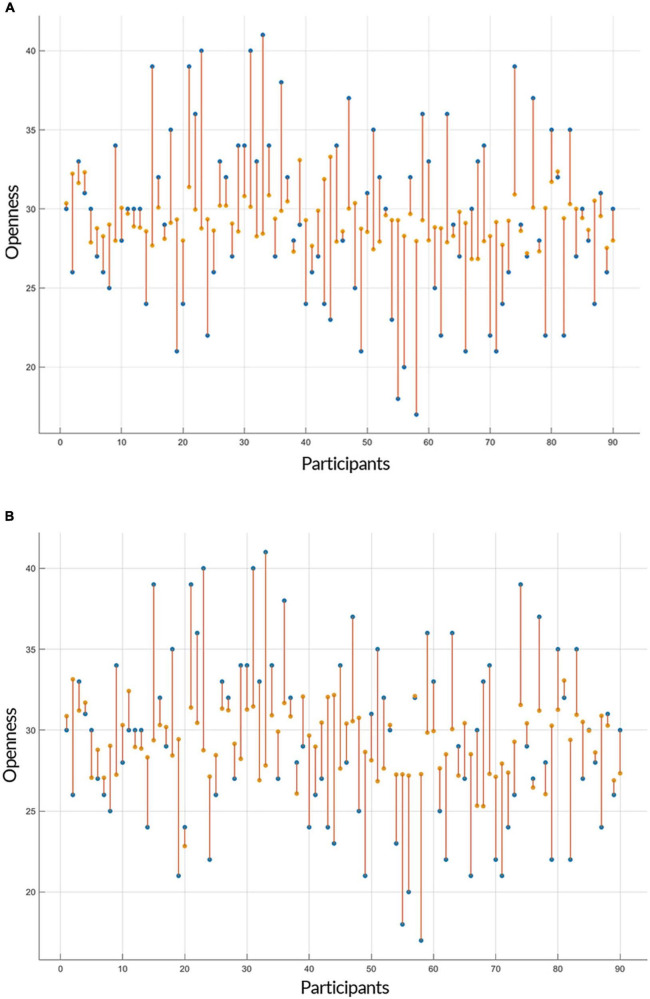
Prediction of openness to experience based on global efficiency (GE) scores. The plots show the result of the prediction of openness to experience scores (y-axis) of each subject (x-axis) by **(A)** Gaussian Process Regression (GPR) and **(B)** linear regression based on the openness to experience and GE scores as inputs. The predicted values are shown in orange and the actual data points are in blue. The orange lines depict the difference between predicted values and the actual observations.

**FIGURE 4:**
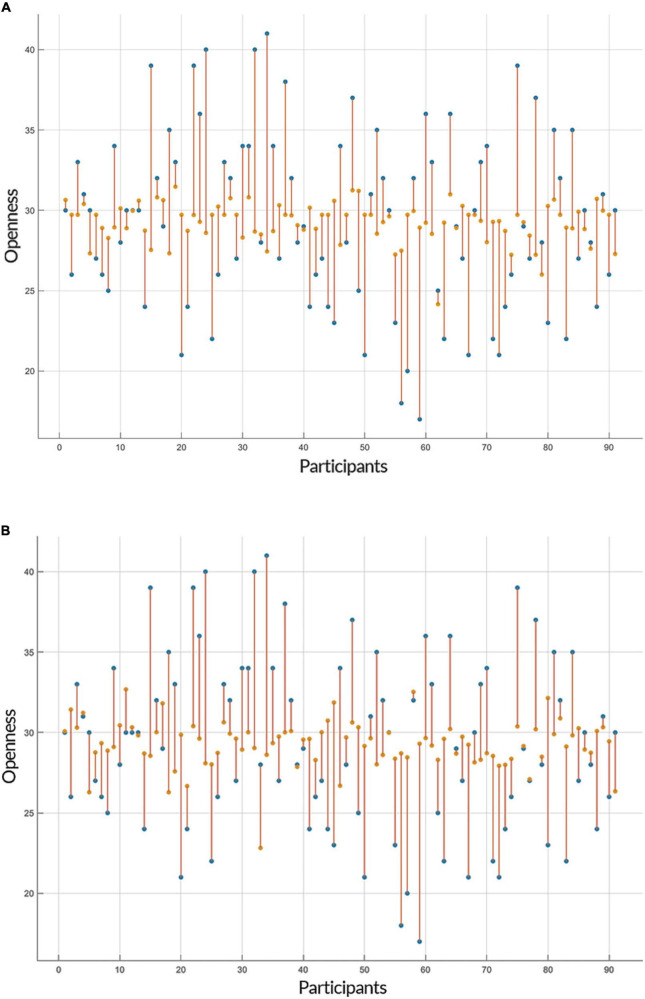
Prediction of openness to experience based on clustering coefficient (CC) scores. Similar to Figure 3, the plots show the result of the prediction of openness to experience scores (y-axis) of each subject (x-axis) by **(A)** Gaussian Process Regression (GPR) and **(B)** linear regression, but this time based on the openness to experience and CC scores as inputs. The predicted values are shown in orange and the actual data points are in blue. The orange lines depict the difference between predicted values and the actual observations.

Both linear and non-linear regression models provided a similar range of accuracy in their predictions of openness to experience based on GE and CC measures. The Root Mean Square Errors (RMSE) of GPR (with an exponential kernel) and linear regression models for both inputs (i.e., GE and CC) were as followed: GE: GPR = 5.534, Linear = 5.334; CC: GPR = 5.418, Linear = 5.346 and the accuracy of models for two predictors (GE and CC) was: GE: GPR = 83.8%, Linear = 84.6%; CC: GPR = 83.7%, Linear = 83.9%. Figures 3, 4 show individual distances between predicted and real values of openness to experience based on CC and GE.

## Discussion

Our goal in the current study was to investigate the relationship between network features of the brain’s structural connectome and openness to experience. Our results showed that there is a significant negative correlation between GE as a measure of integration in the network and openness to experience which means higher openness to experience is associated with a lower capacity of the brain in integrating information from different regions. CC as a measure of network segregation showed an initial significant association with openness but after correcting for multiple comparisons, this association was no longer significant. Then we used linear and non-linear regression models to predict openness to experience based on structural network features and these models predicted the openness scores with a similar range of accuracy.

Most studies that investigated the structural/functional underpinnings of personality have focused on regional brain functions or structure (DeYoung et al., 2010; Xu and Potenza, 2012; Bjørnebekk et al., 2013; Privado et al., 2017; Wang et al., 2018, 2022; Owens et al., 2019; Rodriguez et al., 2019; Avinun et al., 2020). For example, some structural studies reported a positive association between openness to experience and FA values in IFO and ILF (Xu and Potenza, 2012; Privado et al., 2017). Others found a negative correlation between openness to experience and the cortical thickness of the dorsolateral and ventrolateral prefrontal cortex (DLPFC and VLPFC) in both hemispheres as well as a positive association with the area and volume of the left inferior temporal cortex and the volume of the right insula (Owens et al., 2019). Since the involvement of various regions has been suggested, we argued that the neural bases of openness to experience can be better understood by utilizing the graph theoretical analysis as a comprehensive framework to study the structural networks (consisting of many regions). Our result supports this argument as it suggests a negative association between structural integration of the network and openness to experience. According to this result, when structural wiring between brain regions is more efficient (to integrate distinct neural signals), openness to experience is reduced. Hence, our result backs up the idea that adding a network perspective to the investigation of brain structure and function can advance our understanding of personality (Markett et al., 2018; Toschi et al., 2018).

Another possible implication of our result is that it might be suggestive of an overlap between the neurocognitive mechanisms of openness to experience and psychosis. Several studies have shown that psychosis is associated with heightened openness to experience (Fyfe et al., 2008; DeYoung et al., 2012; De Fruyt et al., 2013; Gore and Widiger, 2013; Thomas et al., 2013), and emerging evidence suggest that default mode network and frontoparietal control network are associated to both psychosis proneness and openness to experience (Blain et al., 2019, 2020). We speculate that openness to experience and psychosis might also have similar neural mechanisms at the level of structural connectivity. Schizophrenia as the most prominent form of psychosis is associated with altered structural connectivity, especially reductions in white matter coherence of tracts (Pettersson-Yeo et al., 2011; Cannon, 2015). Some studies found that these alterations result in decreased measures of network integration in the structural connectome (Rubinov and Bullmore, 2013; Drakesmith et al., 2015). The result from our study could be interpreted in relation to the aforementioned studies, as both high openness to experience and psychosis appear to be linked to decreased structural brain network integration. Hence, our findings seem to be in line with the idea that most symptoms of mental disorders exist on a continuum with normal personality traits and typically involve maladaptive forms of normal traits (DeYoung and Krueger, 2018). Further research is needed to investigate the relationship between openness to experience, psychosis, and structural network features.

Although the current study was the first study that investigated the relationship between graph measures gained from structural connectome and openness to experience, previous studies have investigated the relationship between this trait and network features of functional brain networks. Toschi et al. (2018) investigated the relationship between openness to experience and various global and local graph indices including global efficiency and clustering coefficient in a large sample of 818 subjects but did not find any relationship between measures of network integration and openness to experience. On the other hand, Beaty et al. (2016) found a significant positive association between openness to experience and default mode network in two studies with sample sizes of 68 and 86 subjects. While the inconsistency between the results of these studies may be due to their significantly different sample sizes, the incompatibility of their results with our findings might be a consequence of intrinsic features of brain architecture; namely the complex relationship between structural and functional connectivity. Several studies have shown that structural and functional connectivity cannot be associated in a straightforward manner (Uddin, 2013). Although in many cases there are positive correlations between structural and functional connectivity (Straathof et al., 2019), it has long been observed that functional connectivity can exist between regions that are not directly connected with white matter tracts. For example, research on split-brain has shown that even in the absence of transverse fibers, bilateral resting-state functional connectivity stays intact (Uddin et al., 2008; Tyszka et al., 2011).

While numerous research has been done on the association between graph measures of the functional connectome and the five-factor model, few studies have investigated the relationship between graph measures of structural connectome and personality. The lack of straight forward relationship between functional and structural connectome shows the necessity of further research on the relationship between personality and network features of the structural connectome. In addition, given the complex relationship between structural and functional connectivity, future research can explore the relationship between personality traits and structural as well as functional connectome using methods like multi-layer modeling of brain networks (De Domenico, 2017). In a multi-layer network, each layer represents a specific type of information about the system, from neural activity with respect to various tasks, to structural or functional connectivity. Compared with a structural or functional network, a multi-layer network will provide a more accurate representation of the complex organization of the brain in spatial and temporal dimensions and therefore might be able to provide a better account of neurocognitive mechanisms of personality (De Domenico et al., 2013; De Domenico, 2017).

Our study is associated with some limitations. We should emphasize that this study was conducted using a subset of the HCP dataset, and therefore a similar study that uses the whole dataset would be favorable in evaluating our results. Future studies can also evaluate the relationship between openness to experience and *influence measures* (e.g., betweenness centrality) which are important for detecting network hubs that are associated with openness to experience. Furthermore, although HCP data is one of the most reliable open-access data (Van Essen et al., 2013) and other personality studies also benefited from using it (Toschi et al., 2018; Owens et al., 2019), the sample is mainly comprised of young and healthy adults from the United States which can affect the generalizability of our results. Therefore, similar studies need to be done using more diverse samples (regarding age, race, and ethnicity). The last point to be noted is that since outlier removal affected the results, our findings should be interpreted with caution, which again, shows the importance of further research on the relationship between openness to experience and network features of the structural connectome.

In conclusion, our results provided new insights into the relationship between openness to experience and structural brain architecture at the network level. Since the whole-brain network analysis consists of a wide range of cortical and subcortical brain regions, our result may justify the involvement of various distinct regions which came up in previous studies (DeYoung et al., 2010; Xu and Potenza, 2012; Privado et al., 2017; Owens et al., 2019). Specifically, our results showed a significant negative relationship between structural brain network integration and openness to experience. This can suggest that lower integration in structural brain network wiring is associated with higher openness to experience.

## Data availability statement

Publicly available datasets from the Human Connectome Project (HCP) were analyzed in this study. This data can be accessed by registering at the ConnectomeDB website: https://db.humanconnectome.org/.

## Author contributions

NT and AG contributed to the conception and design of the study and wrote the manuscript. NT performed imaging and statistical analysis. Both authors contributed to the article and approved the submitted version.

## Footnotes

1
http://www.mrtrix.org

2
http://www.fmrib.ox.ac.uk/fsl/

3
https://sites.google.com/site/bctnet/
