# Supplementary material for: Integration of structural brain networks is related to openness to experience: A diffusion MRI study with CSD-based tractography
Source: Front Neurosci. 2022 Dec 8;16:1040799. doi: 10.3389/fnins.2022.1040799 (PMC9775296; doi:10.3389/fnins.2022.1040799)
Supplement: Supplementary file 1 [file Data_Sheet_1.docx]

Supplementary Material

**Supplementary Table 1.** The list of subjects’ IDs whose data were analyzed in the study. The data is available in the Human Connectome Project (HCP) database.

| Subject’s ID | Subject’s ID | Subject’s ID |
| --- | --- | --- |
| 102614 | 118023 | 180129 |
| 102715 | 118124 | 180230 |
| 103010 | 118528 | 180432 |
| 103212 | 118730 | 180533 |
| 103414 | 118932 | 180735 |
| 103515 | 119025 | 180836 |
| 103818 | 121416 | 181131 |
| 104012 | 121618 | 181232 |
| 104416 | 121719 | 181636 |
| 105115 | 121921 | 182032 |
| 105216 | 122317 | 182436 |
| 105620 | 122620 | 182739 |
| 105923 | 123117 | 182840 |
| 106016 | 123521 | 183034 |
| 106319 | 136126 | 183337 |
| 106521 | 136631 | 183741 |
| 106824 | 136732 | 200008 |
| 107018 | 136833 | 200109 |
| 107321 | 137027 | 200210 |
| 107422 | 137229 | 200311 |
| 108020 | 137532 | 200513 |
| 108121 | 137633 | 200614 |
| 108222 | 137936 | 201111 |
| 108828 | 138130 | 201414 |
| 109830 | 138332 | 201515 |
| 110007 | 138837 | 202719 |
| 110411 | 139233 | 203418 |
| 110613 | 139435 | 203923 |
| 111009 | 139637 | 204218 |
| 111211 | 139839 | 206323 |
| 111413 | 140117 | 208024 |
| 111514 | 140824 | 223929 |
| 111716 | 141119 |  |
| 112112 | 141422 |  |

**Supplementary Table 2.** The association between openness to experience scores and two graph measures of global efficiency and clustering coefficient using linear regression without outlier removal.

| Graph measure | Pearson's r | R^2^ | Adjusted R^2^ | RMSE | Standard Error | F-statistic | p-value | Number of subjects |
| --- | --- | --- | --- | --- | --- | --- | --- | --- |
| *Global efficiency* | -0.1547 | 0.023 | 0.014 | 6.02 | 7.901 | 2.4 | 0.124 | 100 |
| *Clustering coefficient* | -0.1252 | 0.015 | 0.005 | 6.05 | 78.32 | 1.54 | 0.217 | 100 |
